# Ultrasound-Induced Release Profile of Nimodipine from Drug-Loaded Block Copolymers after Singular vs. Repeated Sonication: In Vitro Analysis in Artificial Cerebrospinal Fluid

**DOI:** 10.3390/brainsci14090912

**Published:** 2024-09-10

**Authors:** Katja Döring, Swetlana Sperling, Milena Ninkovic, Heinrich Lanfermann, Frank Streit, Andreas Fischer, Veit Rohde, Vesna Malinova

**Affiliations:** 1 Department of Neurosurgery, University Medical Center Göttingen, 37075 Göttingen, Germany; doering.katja@mh-hannover.de (K.D.); swetlana.sperling@med.uni-goettingen.de (S.S.); milena.ninkovic@med.uni-goettingen.de (M.N.); veit.rohde@med.uni-goettingen.de (V.R.); 2 Department of Interventional and Diagnostic Neuroradiology, Hannover Medical School, 30625 Hannover, Germany; lanfermann.heinrich@mh-hannover.de; 3 Department of Clinical Chemistry, University Medical Center Göttingen, 37075 Göttingen, Germany; frank.streit@med.uni-goettingen.de (F.S.); andreas.fischer@med.uni-goettingen.de (A.F.); 4 Department of Neurosurgery, Georg-August-University, Robert-Koch-Straße 40, 37075 Göttingen, Germany

**Keywords:** drug release profile, nimodipine, nanodrug

## Abstract

Objective: Nimodipine still represents a unique selling point in the prevention of delayed cerebral ischemia (DCI) following aneurysmal subarachnoid hemorrhage (aSAH). Its intrathecal effect is limited by a low oral bioavailability, leading to the development of nanocarrier systems to overcome this limitation. This study investigated the ultrasound-induced release profile of nimodipine from drug-loaded copolymers in artificial cerebrospinal fluid (CSF) within 72 h after a singular versus repeated sonication. Methods: Pluronic^®^ F127 copolymers (Sigma-Aldrich, Taufkirchen, Germany)were loaded with nimodipine by direct dissolution. Spontaneous and on-demand drug release by ultrasound (1 MHz at 1.7 W/cm^2^) was determined in artificial cerebrospinal fluid using the dialysis bag method. Nimodipine concentrations were measured at predefined time points within 72 h of sonication. Results: Spontaneous release of nimodipine was enhanced by ultrasound application with significantly increased nimodipine concentrations two hours after a repeated sonication compared to a singular sonication (median 1.62 vs. 17.48 µg/µL, *p* = 0.04). A further trend was observed after four hours (median 1.82 vs. 22.09 µg/µL, *p* = 0.06). There was no difference in the overall nimodipine concentrations between the groups with a singular versus repeated sonication (357.2 vs. 540.3 µg/µL, *p* = 0.60) after 72 h. Conclusions: Repeated sonication resulted in an acceleration of nimodipine release from the drug-loaded copolymer in a CSF medium. These findings confirm the proof of principle of an on-demand guidance of nimodipine release from nimodipine-loaded nanodrugs by means of ultrasound, which suggests that evaluating the concept in an animal model may be appropriate.

## 1. Introduction

Delayed cerebral ischemia (DCI) is a common complication of aneurysmal subarachnoid hemorrhage (aSAH) [1]. The pathophysiology of DCI has been intensively investigated in the past years revealing a multifactorial pathogenesis behind this phenomenon [2]. Despite the continuously growing knowledge regarding the pathophysiology, the treatment options for DCI remain limited [3]. While several drugs have been shown to effectively reverse cerebral vasospasm in patients with aSAH, nimodipine is the only drug that was able to improve the patients’ outcome as well [4]. The highest level of evidence exists for the oral administration of nimodipine with six single doses per day [5]. However, first-pass metabolism resulting in an oral bioavailability of only 3–30% limits the intrathecal effect of nimodipine. Additionally, nimodipine-induced side effects after systemic application often led to a reduction or discontinuation of the treatment with nimodipine in clinical practice [6,7]. Addressing these limitations, direct intrathecal nimodipine administration has gained scientific and clinical interest [8]. Nanotechnology is an emerging field of pharmacology that has opened new avenues for direct drug delivery to the site of action [9,10], enabling higher local drug concentrations while circumventing the side effects of the systemic drug administration at the same time. A wide range of synthetic nanostructures (solid lipid nanoparticles, liposomes, nanostructured lipid carriers, nanoshells, quantum dots, and superparamagnetic nanoparticles) has been developed in recent years that can be modulated in size, shape, and surface chemistry and hence provide new solutions for drug delivery [9]. Nanocarriers play an important role in oncology, facilitating the controlled release of anticancer drugs [10].

Polymeric block copolymers consisting of hydrophilic and hydrophobic units, with a hydrophobic core protected by the surrounding hydrophilic chains in aqueous solution, have been already established and proven to be ideal drug carriers for hydrophobic substances such as nimodipine [11,12]. The weak conjugation between the copolymer and the water-insoluble molecule nimodipine is based on hydrophobic interactions, hydrogen bonding and van der Waals forces [13,14]. Several studies on nimodipine-loaded micro- and nanoparticles have been already conducted which have demonstrated a sustained drug release over time immediately after intrathecal administration of drug-loaded nanocarriers [15,16]. In a previous study, we developed a nimodipine-loaded nanodrug and demonstrated a successful on-demand drug release induced by ultrasound [17]. A significantly increased drug release was achieved after a singular sonication. The findings of our previous study gave rise to the question, ‘can the release of nimodipine be potentiated by repeated sonications?’ In this study, the nimodipine release profile from the nanodrug was explored after repeated sonication and compared to singular sonication in an artificial cerebrospinal fluid (CSF) medium to assess the feasibility of this concept for an upcoming evaluation in animal studies.

## 2. Materials and Methods

The in vitro experimental setup included three steps: 1—preparation of Pluronic^®^ F-127 (BASF Corporation, Florham Park, Morris, NJ, USA) block copolymers loaded with nimodipine, 2—measurement of the spontaneous continuous release of nimodipine from the Pluronic^®^ F-127 block copolymers, and 3—measurement of the ultrasound-induced release of nimodipine from the Pluronic^®^ F-127 block copolymers after singular and repeated sonication. Pluronic^®^ F-127 block copolymers were used as nanocarriers without further purification. Pluronic^®^ F-127 is a triblock copolymer of polyethylene oxide and polypropylene oxide (PEO-PPO-PEO) with a molecular weight of 12,600 Da and a hydrophilic–lipophilic balance (HLB) of 22 (all data from the manufacturer).

### 2.1. Artificial Cerebrospinal Fluid

Artificial CSF was used to analyze the release profile of nimodipine from drug-loaded Pluronic^®^ F-127 copolymers in a CSF-like medium. Artificial CSF acts as a biological buffer, providing a vital environment by maintaining homeostasis, osmolarity and pH at physiological levels and is commonly used as a laboratory chemical, not only for in vitro but also for in vivo applications. To prepare 1000 mL of artificial CSF solution, 500 mL of Base A was added to a further 500 mL of Base B. Base A is first oxygenated for 10 min; then, 500 mL of Base B is slowly added. The artificial CSF solution prepared in this way is enriched with oxygen throughout its use. With an oxygen enrichment of 95% O_2_ and 5% CO_2_ (carbogen), the pH is 7.4 (all data from the manufacturer).

### 2.2. Preparation of Nimodipine-Loaded Pluronic^®^ F-127 Block Copolymers

Pluronic^®^ F-127 copolymers loaded with nimodipine were prepared using the direct dissolution method as previously described by Sotoudegana et al. [18]. The preparation involved the following steps: Briefly, 2 mg of nimodipine powder (Sigma-Aldrich Chemical Company, St. Louis, MO, USA) and a defined amount of Pluronic^®^ F-127 (5%), were added simultaneously to 10 mL of DI at a stirring frequency of 100 U/mL. The suspension was then mixed at 100 rpm for 3 h at room temperature (25 °C). The precipitated nimodipine was separated from the micelle suspension by filtration (pluriStrainer^®^ filter with a mesh size of 1 µm, pluriSelect^®^ Life Science, Leipzig, Germany). The preparation process of nimodipine-loaded Pluronic^®^ F127 block copolymers was reported in detail in an article previously published by our research group [19]. The size of the nimodipine-loaded block copolymers was 122.4 ± 12.3 as measured by transmission electron microscope. The nimodipine-loaded block copolymers had a spherical form with a smooth surface. The size and morphology of the nimodipine-loaded block copolymers remained stable for up to three months [19]. In this previous work, the entrapment efficacy, and the percentage drug load of the nimodipine-loaded block copolymers were evaluated using three different Pluronic^®^ F127 concentrations (5%, 10% and 15%). In this study, the nimodipine-loaded block copolymers with a 5% Pluronic^®^ F127 concentration were used with an entrapment efficacy of 46% and a percentage drug load of 59.58% [19].

### 2.3. Drug Release from Drug-Loaded-Pluronic^®^ F 127 Block Copolymers

The release of nimodipine from the nimodipine-loaded block copolymers in artificial CSF was evaluated in two steps: 1—spontaneous drug release without external influence and 2—controlled drug release induced by a singular and repeated ultrasound application. The spontaneous and ultrasound-induced release profile setup was repeated five times for every experimental setup (spontaneous, one sonication and two sonications).

### 2.4. Spontaneous Nimodipine Release without External Influence

**Figure 1:**
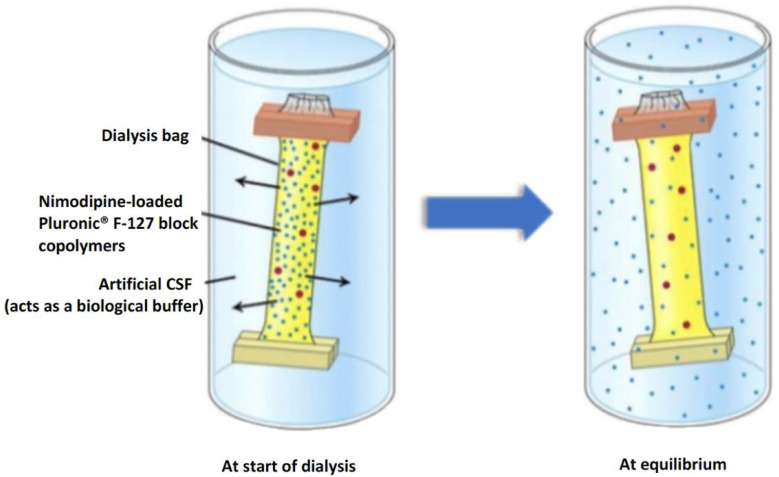
Dialysis bag method with the status at the beginning and after the nimodipine release from the nanodrug.

The in vitro drug release profile of nimodipine from the Pluronic^®^ F 127 copolymers in artificial CSF was evaluated using the dialysis bag method (Figure 1). For this purpose, the dialysis bags (Spectrum™ Labs Spectra/Por™ 6 3500 D MWCO, Fisher Sientific, Schwarte, Germany) were first soaked in deionized water for 24 h and stored in a cool place at 4 °C until use. For experimental conversion, 10 mL of the nimodipine-loaded micellar solution was added to the dialysis bag. The respective ends of the bags were clamped as intended and placed in 200 mL artificial CSF solution. The whole set-up was stirred at 36.5 °C for 72 h at 100 rpm. At predetermined time points (0, 5, 15 and 30 min and 2, 4, 24, 48 and 72 h) an aliquot of 300 µL was taken from the dissolution medium.

**Figure 2:**
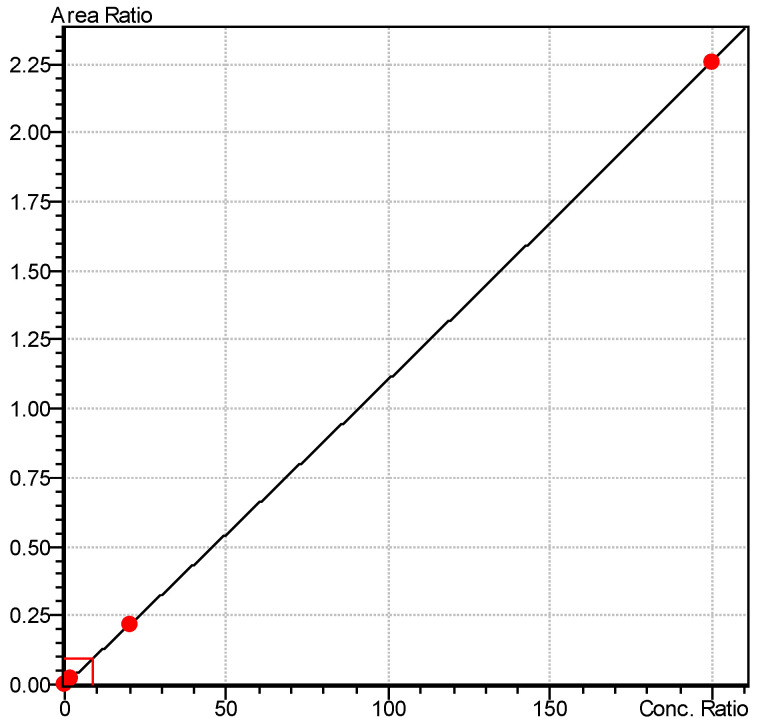
Calibration curve 0.0108x + 0, r = 0.9999693.

**Figure 3:**
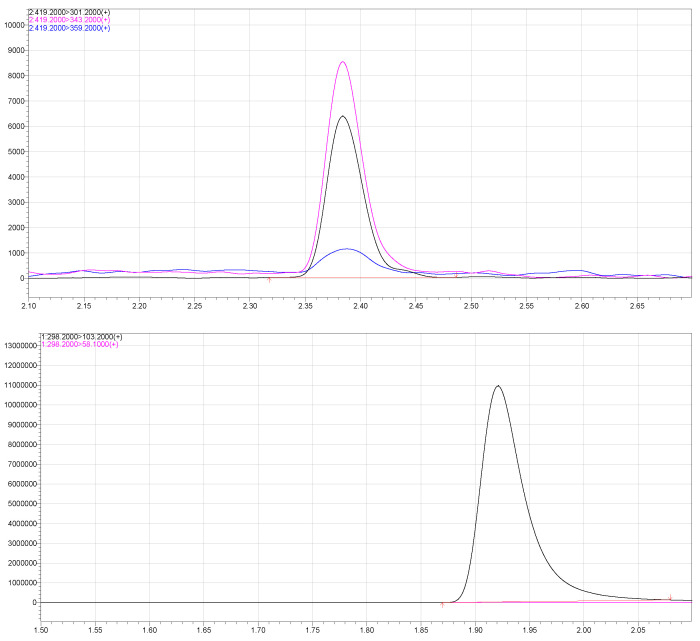
Ion chromatograms of nimodipine (Cal1 0.2 µg/L) and internal Standard D3-trimipramine.

The samples obtained were then immediately frozen at −20 degrees Celsius without further dilution until subsequent analysis using a mass spectrometer. The amount of drug released into the medium was calculated from a calibration curve. A hydroalcoholic solution of nimodipine (Nimodipine Carinopharm, Carinopharm GmbH, Elze, Germany) at a concentration of 0.2 mg/mL was used as a control. For each condition, the analysis was performed five times to determine the mean values and to ensure reproducibility. A Nexera X2 UHPLC, Shimadzu, Duisburg, Germany (Ultra High-Performance Liquid Chromatography) connected to a LCMS-8050 mass spectrometer (Shimadzu, Kyoto, Japan) equipped with an electrospray ion source was used for the determination of nimodipine concentration. A sample volume of 0.1 µL was injected into a Halo 50 × 4.6 × 2.7 µm (Advanced Material Technologies, MZ Analysentechnik, Mainz, Germany). A sharp gradient with mobile phase A (5% ammonium acetate) and mobile phase B (methanol) was used as follows: Initial conditions were 3% B with a flow rate of 0.9 mL/min. Then, 3% B was held for 0.02 min, a linear gradient towards 50% B was used up to 0.8 min and a linear gradient to 95% B was used until 2 min. Column was washed for 0.4 min with 95% B and equilibrated with 3% B from 2.5 to 3 min. For quantification, the MRM (Multiple Reaction Monitoring) transitions 419.2/301.0, CE-22.0, as the quantifier and 343.2, CE-12.0, as a qualifier for nimodipine (rt = 1.27 min) and m/d 3z 295.0/100.0 for internal standard D3-trimipramine (rt 0.65 min) were monitored. Linearity was established in the range of 0.2–200.0 µg/L (0.0108x + 0, r = 0.9999693) (Figure 2 and Figure 3). Within a run, at QC1 (Quality Control 1), 10.0 µg/L CV (cyclic voltammetry) of 0.868% was found, and at QC2 (Quality Control 2), 100.0 µg/L, a CV of 0.983% was found. The CV of 4.03% was found at the LLOQ (Lower Limit of Quantification) of 0.2 µg/L. Between runs, CV was 11.6% for QC1 and 6.7% for QC2.

### 2.5. Ultrasound-Induced Nimodipine Release

**Figure 4:**
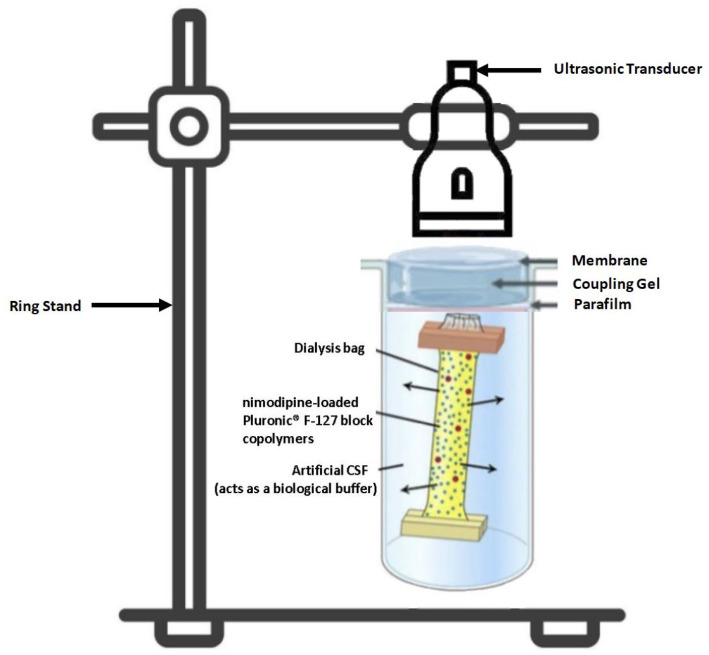
Experimental setup during the ultrasound-induced drug release.

For an induced drug release, low frequency ultrasound waves were applied either one or twice using PHYSIOSON-Expert (Physiomed^®^, Paderborn, Germany). For the experimental setup, two batches of five samples each were sonicated at different intensities. The experimental setup is demonstrated in Figure 4. While the technical variables remained the same (high-intensity continuous ultrasound with a frequency of 1 MHz and an intensity of 1.7 W/cm^2^), the time variable (t) was modulated: the ultrasound treatment was performed for either 30 or 60 s. As described above, 10 mL of each of the different concentrations of the nimodipine-loaded micelle solution were filled into the dialysis bags and added to 200 mL of artificial CSF. The ultrasound probe, which was previously wetted with ultrasound gel, was positioned on the dialysis bag so that the ultrasound probe touched the surface of the CSF medium in the beaker. The ultrasound treatment was then performed and an aliquot of 300 µL was taken from the dissolution medium at the same predetermined times (0, 5, 15 and 30 min and 2, 4, 24, 48 and 72 h) under static conditions analogous to the measurement of the spontaneous drug release profile described above (36.5 °C for 72 h at 100 rpm). Again, the samples were frozen at minus four degrees Celsius until they were analyzed in a mass spectrometer. The technique used is like that described above (see Section 2.4). As mentioned above, each condition was run five times to determine the mean.

### 2.6. Statistical Analysis

Statistical analysis was performed using GraphPad Prism (version 9.0, GraphPad Software, San Diego, CA, USA). A *p*-value of <0.05 was used as the significance level. All data are expressed as mean ± SD or median with 95% confidence interval (CI) and/or interquartile range (IQR). Classical ANOVA analysis was used for subgroup comparisons.

## 3. Results

### 3.1. Spontaneous Release Profile of Nimodipine from Nimodipine-Loaded Pluronic^®^ F 127 Block Copolymer

During the first two to four hours, a continuous, slow, and shallow drug release was observed. This was followed by a 6-fold increase in nimodipine concentration after 24 h. A further doubling of the release rate occurred between 48 and 72 h.

### 3.2. Ultrasound-Induced Drug-Release after Singular vs. Repeated Sonication

A summary of concentrations of nimodipine released spontaneously without external influence (control group), as well as that released after ultrasound application with singular and repeated sonication is given in Table 1.

An increase in the released nimodipine concentration was seen already 30 min after sonication with a potentiation of the effect after repeated sonication. The median nimodipine release without external influence, i.e., concentration of spontaneously released nimodipine after 30 min was 0.24 µg/µL. That increased to 0.60 µg/µL after one sonication, and reached 14.8 µg/µL after repeated sonication, but the difference did not reach statistical significance.

**Figure 5:**
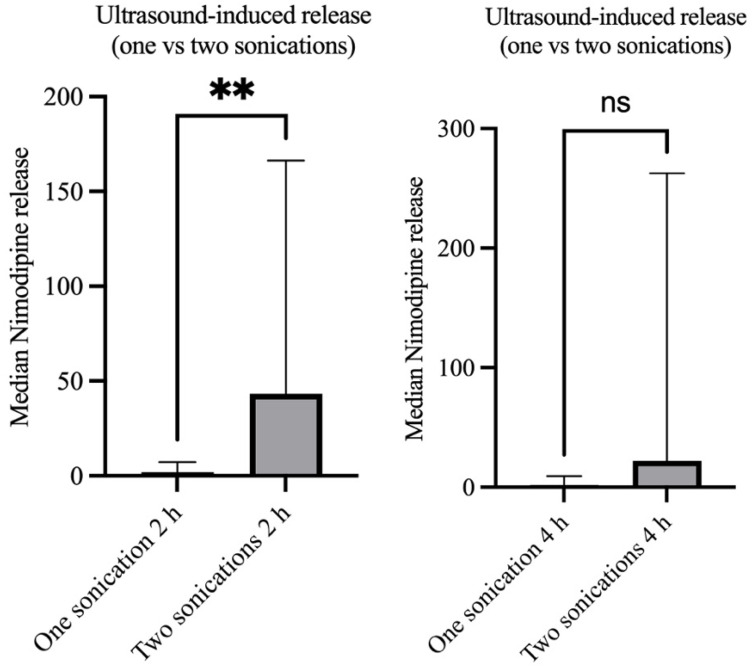
Nimodipine release profile two and four hours after a singular versus repeated sonication showing a significantly increased nimodipine concentration in the group with repeated sonication after two hours, but without a significant difference between the two groups after four hours. “**” states a statistically significant difference. “ns” states non-significant differences.

**Figure 6:**
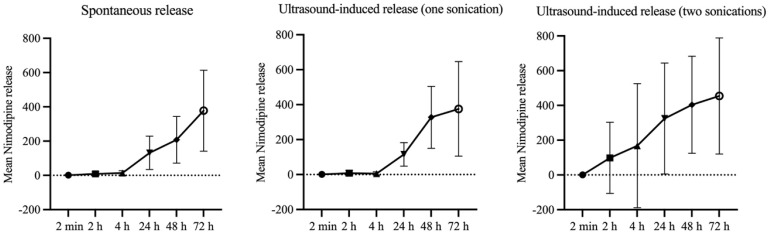
The time course of spontaneous and on-demand nimodipine release profile by singular and repeated sonication over a period of 72 h.

A direct comparison of the groups with singular sonication and repeated sonication showed a significantly increased early nimodipine release within the first two hours in the group with repeated sonication (median nimodipine concentration 1.62 vs. 17.48 µg/µL, *p* = 0.04). A further trend was seen at 4 h in the group with repeated sonication (median nimodipine concentration 1.82 vs. 22.09 µg/µL, *p* = 0.06) (Figure 5). A comparison of the two groups after 72 h shows no difference in released concentrations (median nimodipine concentrations 357.2 vs. 540.3 µg/µL, *p* = 0.60), indicating that drug release increases early after sonication and returns to baseline in the long term (Figure 6).

## 4. Discussion

In this in vitro study, the nimodipine release from a drug-loaded nanocarrier could be successfully enhanced through repeated sonications. These findings proofed the concept of an on-demand drug release by applying ultrasound. A time-dependent increase in nimodipine concentrations was measured within the first two hours after sonication, following a gradual return to baseline again starting four hours after sonication. This allowed a temporary, on-demand increase in nimodipine concentration within the CSF by means of ultrasound, which was a prerequisite for the implementation of this concept in clinical practice. Because previous studies showed that a reversal of angiographic vasospasm does not necessarily result in a better outcome, a neuroprotective effect of nimodipine is deemed to be responsible for the positive impact of nimodipine on the outcome [20,21,22]. Currently, nimodipine is used in clinical practice with prophylactics (i.e., prevention of cerebral vasospasm) as well as therapeutic interventions (treatment of manifested cerebral vasospasm causing neurological deficits and/or cerebral perfusion deficits). The nimodipine-loaded nanodrug presented in our study with a continuous spontaneous drug release as well as an increased on-demand release through sonication seems to be suitable for both purposes.

### 4.1. Advantages and Limitations of Systemic Administration Routes for Nimodipine

A meta-analysis conducted by Geraldini et al. in 2022 showed that both oral and intravenous nimodipine were effective in preventing unfavorable outcomes and DCI, but had no influence on mortality [23]. Another meta-analysis published in 2023, which included nine randomized controlled trials, demonstrated no statistically significant difference between intravenous and enteral administration in terms of mortality, DCI, delayed ischemic neurological deficits and outcome [24]. However, the area under the cumulative ranking curve showed a trend for enteral administration to be first, intravenous administration to be second, and placebo to be last in terms of mortality, occurrence of DCI, and poor outcomes [24]. In a more recently published retrospective, multicenter, observational cohort study conducted in 21 hospitals across North America, Mahmoud et al. assessed the extent to which different nimodipine formulations and routes of administration were associated with the safety and efficacy of nimodipine in aSAH [25]. While the administration of nimodipine in liquid form was independently associated with a higher prevalence of diarrhea, the withdrawal of liquid from nimodipine capsules prior to administration was significantly associated with a higher prevalence of nimodipine dose reduction or discontinuation due to hypotension. Crushing the tablets and withdrawing the liquid from the capsules at the bedside before administration were associated with an increased likelihood of DCI [25]. In an observational cohort study, Rass et al. recorded hemodynamic responses in patients with SAH receiving prophylactic nimodipine with either oral or intravenous administration [26]. Hemodynamic responses were assessed within the first hour after the start of nimodipine therapy. It was found that 30% of patients experienced a reduction in blood pressure of more than 10% immediately after the start of nimodipine infusion, with the maximum effect occurring after 15 min [26]. Approximately half of these patients required an immediate increase in norepinephrine, and a further 10% required colloids within one hour of the start of the nimodipine infusion to counteract a further drop in blood pressure [26]. The situation was different with oral nimodipine administration, where significant reductions in blood pressure of >10% occurred later and less frequently—with a consequent increase in the use of noradrenaline. Changes in mean arterial blood pressure (MAP), cerebral perfusion pressure (CPP), cerebral tissue oxygen tension (pbtO_2_) and cerebral metabolism after oral administration of nimodipine were analyzed in a retrospective study using mixed linear models [27]. Oral administration of nimodipine was shown to reduce MAP, leading to a reduction in cerebral perfusion and oxygenation [27]. However, this study is limited by the small number of cases and the retrospective study design. Furthermore, nimodipine, as a dihydropyridine calcium channel antagonist acts on countless cell types throughout the body and has probably more complex mechanisms of action than simply preventing cerebral vasoconstrictions [28].

### 4.2. Advantages and Limitation of Local Nimodipine Administration

Advances in the development of alternative administration pathways for nimodipine have reignited interest in refining its potential therapeutic use. A site-specific, sustained-release administration may increase drug concentrations at the site where it is most needed, while avoiding additional adverse effects associated with systemic hypotension. Local drug administration, i.e., pellet-based therapeutics placed around the basal cerebral arteries during aneurysm clipping with continuous release of the calcium antagonist nicardipine, have been shown to be safe [29,30,31]. Furthermore, local drug administration was associated with less hypotension, led to significantly higher drug concentrations at the target organ, and resulted in a less frequent occurrence of cerebral vasospasm [20,30]. However, pellet-based therapeutics can be used only in surgically treated patients, which limits their use in patients undergoing endovascular coiling to repair ruptured aneurysms [32]. Accordingly, the idea of a new platform for the local administration of nimodipine with delayed release using polymers is maturing. Several studies on the intrathecal administration of calcium channel blockers bound to polymers have already been published [8,18,33,34]. An initial pharmacokinetic evaluation showed that the release of nimodipine after administration consisted of an initial surge followed by a sustained release over 21 days [8].

Based on these encouraging results, the PROMISE (Prolonged Release nimodipine microparticles after Subarachnoid hemorrhage) trial was initially designed in 2015 as a single-center, open-label, non-randomized, dose-escalating Phase I study to evaluate the efficacy, safety and tolerability of the intracisternal administration of EG-1962 (nimodipine in a biodegradable polymer suspended in hyaluronic acid administered as one intraventricular injection that releases nimodipine into the subarachnoid space for at least 21 days) in patients undergoing surgical treatment for aSAH [18]. At the same time, Hänggi et al., who are also the principal investigators and authors of the PROMISE study, initiated the NEWTON study (Nimodipine microparticles to Enhance recovery While reducing Toxicity after subarachnoid hemorrhage) [33]. In contrast to PROMISE, NEWTON is designed as a multicenter, controlled, randomized, open-label, dose-escalation study to evaluate the safety, tolerability, and pharmacokinetics of EG-1962 and nimodipine in patients with aneurysmal SAH, and has already demonstrated efficacy in a Phase 2 study [33]. Across the board, EG-1962 was considered safe and well tolerated. In addition, the group treated with EG-1962 showed a lower rate of DCI—correspondingly, the need for rescue therapy was also lower. Overall, the rate of favorable clinical outcomes was higher in the EG-1962-treated group than in the conventionally treated group [33]. The limitation of these nanodrugs was the lack of ability to externally influence the drug release on demand, which was addressed in our study. With this in vitro study, we were able not only to demonstrate an on-demand increase in drug release through singular sonication, but also to show the possibility of further modifying the drug release by means of repeated sonication, which opens the door for the direct guidance of drug release. In addition to the continuous prophylactic release to prevent vasospasm, we were also able to demonstrate and prove an on-demand release using low-frequency ultrasound, opening up the possibility of on-demand therapeutic intervention in addition to highly effective local prophylaxis.

### 4.3. Limitations of the Study

As in vitro experiments were conducted, the study does not allow conclusions regarding the effects of the nanodrug in an in vivo scenario. The findings of this experimental study represent the basis for planning and conducting in vivo evaluations of the concept in animal models. Future studies are also needed to shed light on the mechanism involved in ultrasound-mediated drug release from nanodrugs, because this was not the subject of our study and the mechanisms behind it remain unclear. Further experiments are required to answer the question, ‘how many sonications are needed to release all of the nimodipine from the nanodrug?’ Despite the limitations, the results of our study encourage the further evaluation of the concept in animal models and can be seen as a solid basis for planning future experiments.

## 5. Conclusions

The data obtained support the successful results of our previously published study of the nanocarrier system of nimodipine-loaded Pluronic^®^ F-127 copolymers in an artificial CSF medium. The experiments presented here confirm a further significant on-demand increase in nimodipine release after repeated sonications. These results support the concept of ultrasound-controlled treatment of cerebral vasospasm by increasing the nimodipine release from a nimodipine-loaded nanodrug on-demand by applying ultrasound.

Further evaluation in animal studies or other in vivo environments is required to further explore this promising concept, the determinants of which have now been tested and proven several times.

## Figures and Tables

**Table 1:** Release profile of nimodipine; spontaneous, after one sonication and after repeated sonication.

Experimental Settings	Mean	SD	Median	95% CI	IQR
Control group without sonication					
Control group 2 min	1.439	3.010	0.20	0.2–7.58	0.20–5.40
Control group 15 min	3.588	8.185	0.20	0.19–20.30	0.19–5.40
Control group 30 min	4.867	6.757	0.24	0.2–17.6	0.20–9.12
Control group 2 h	8.547	11.60	2.91	0.2–28.85	0.46–19.11
Control group 4 h	13.35	13.67	10.88	0.2–36.43	1.01–24.42
Control group 24 h	131.6	96.90	118.3	0.2–246.6	51.93–239.6
Control group 48 h	207.7	136.2	219.0	0.2–382.40	104.1–305.7
Control group 72 h	377.7	236.0	355.3	0.2–681.9	228.3–597.1
Treatment group with a singular sonication					
One sonication 2 min	1.243	1.539	0.546	0.24–4.21	0.29–2.24
One sonication 15 min	1.303	2.291	0.454	0.20–5.97	0.21–1.88
One sonication 30 min	1.226	1.687	0.607	0.20–4.63	0.35–1.83
One sonication 2 h	8.296	16.31	1.624	0.20–41.50	0.86–13.18
One sonication 4 h	5.606	9.520	1.822	0.20–24.83	0.61–9.35
One sonication 24 h	115.8	67.53	124.2	0.20–200.4	70.40–164.1
One sonication 48 h	327.5	176.8	360.0	0.43–506.2	220.3–460.2
One sonication 72 h	375.8	270.1	357.2	0.20–754.4	145.5–632.5
Treatment group with repeated sonication					
Repeated sonication 2 min	0.532	0.544	0.27	0.20–1.58	0.20–0.90
Repeated sonication 15 min	1.292	2.414	0.20	0.20–6.20	0.20–2.11
Repeated sonication 30 min	98.72	177.4	14.85	0.51–364.7	3.82–277.5
Repeated sonication 2 h	98.25	204.9	17.48	6.15–516.3	8.10–146.6
Repeated sonication 4 h	168.0	357.1	22.09	0.64–896.1	11.96–262.7
Repeated sonication 24 h	395.6	461.7	204.0	0.65–1226	80.44–780.6
Repeated sonication 48 h	403.7	279.2	422.8	0.54–727.1	166.7–651.9
Repeated sonication 72 h	454.5	334.3	540.3	0.96–736.3	102.5–720.6

SD = standard deviation, CI = confidence interval, IQR = interquartile range.

## Data Availability

All relevant data and materials are presented in the manuscript.
